# Nonlinear Variability of Body Sway in Patients with Phobic Postural Vertigo

**DOI:** 10.3389/fneur.2013.00115

**Published:** 2013-08-15

**Authors:** Roman Schniepp, Max Wuehr, Cauchy Pradhan, Sergej Novozhilov, Siegbert Krafczyk, Thomas Brandt, Klaus Jahn

**Affiliations:** ^1^Department of Neurology, University of Munich, Munich, Germany; ^2^German Center for Vertigo and Balance Disorders, University of Munich, Munich, Germany; ^3^Institute of Clinical Neurosciences, University of Munich, Munich, Germany

**Keywords:** stance, psychogenic, vertigo, sample entropy, DFA, scaling exponent

## Abstract

**Background:** Subjective postural imbalance is a key symptom in the somatoform phobic postural vertigo (PPV). It has been assumed that more attentional control of body posture and / or co-contraction of leg muscles during standing is used to minimize the physiological body sway in PPV. Here we analyze nonlinear variability of body sway in patients with PPV in order to disclose changes in postural control strategy associated with PPV.

**Methods:** Twenty patients with PPV and 20 age-matched healthy subjects (HS) were recorded on a stabilometer platform with eyes open (EO), eyes closed (EC), and while standing on a foam rubber with eyes closed (ECF). Spatio-temporal changes of the center of pressure (CoP) displacement were analyzed to assess the structure of postural variability by computing the scaling exponent α and the sample entropy (SEn) of the time series.

**Results:** With EO on firm ground α and SEn of CoP displacement were significantly lower in patients (*p* < 0.001). For more difficult conditions (EC, ECF) postural variability in PPV assimilated to that of HS.

**Conclusion:** Postural control in PPV patients differs from HS under normal stance condition. It is characterized by a reduced scaling behavior and higher regularity. These changes in the structure of postural variability might suggest an inappropriate attentional involvement with stabilizing strategies, which are used by HS only for more demanding balance tasks.

## Introduction

Phobic postural vertigo (PPV) has been described as a syndrome of subjective postural and gait instability in combination with a non-rotational vertigo or dizziness, mainly affecting patients with an obsessive-compulsive personality (1). PPV is one of the primary and secondary somatoform dizziness syndromes (2–4) and is also termed visual vertigo syndrome (5) or chronic subjective dizziness (6). As one of the most frequent causes of chronic dizziness, it has a high impact on functioning and quality of life (2, 7). The diagnostic criteria mainly comprise anxiety related symptoms in combination with normal otoneurological testing (1). However objective tests for the positive diagnosis of PPV are not available.

Body sway recording has been suggested to be of value in PPV (8, 9). Postural control is commonly assessed by analyzing amplitude parameters such as sway amplitude and sway path length. However, these parameters do not reveal information about the variability of sway, which is an important feature of motor control (10). Postural behavior is characterized by strong nonlinearities due to elastic and damping properties of muscles and nonlinear feedback control (11, 12). Analytic approaches from nonlinear systems theory seem promising for describing the structure of postural variability and have been used in several studies (13–16). Moreover, it has been suggested that nonlinear measures more reliably quantify body sway than traditional amplitude-related measures (17).

It has been hypothesized that altered postural behavior in patients with PPV is caused by an inappropriate postural strategy in which patients exert extensive supraspinal control in particular during normal stance conditions (18–20). In contrast, healthy balance control is mainly achieved by automated mechanisms not involving conscious control (21). Increased alertness is required for unusual and non-adapted balance tasks, which bare the risk of falls. Attentional postural control influences the structure of body sway variability (14), which can be quantified by the analysis of the scaling exponent α and the sample entropy (SEn). Both measures provide information related to the underlying motor control strategies governing postural stability. The scaling exponent α examines the presence of long-range correlations within the time series of the center of pressure (CoP) trajectories. The presence of long-range correlations implies that the postural sway behavior at any given point of time depends on the global history of the CoP time series: the stronger the long-range correlations within CoP time series, the more constrained the overall postural sway behavior (22). In addition, SEn quantifies the regularity or predictability of the CoP time series.

The purpose of this study was to investigate stance behavior in patients with PPV by analyzing the structure of postural variability, which has been shown to reveal motor control strategies governing postural control. The major question of the study was whether there are changes in the structure of body sway variability in patients with PPV that may indicate that these patients apply postural control strategies that are inappropriate with respect to normal balance conditions. Moreover, we aimed to see whether postural strategies change under more demanding balance tasks, like standing with eyes closed (EC) or on a foam rubber.

## Materials and Methods

### Subjects

Twenty patients with PPV (14 females; mean age 38.4 ± 13.8 years; mean height: 1.75 ± 0.11 m; mean weight: 72.4 ± 8.3 kg) and 20 age-matched asymptotic healthy subjects (HS) (8 females; mean age 37.1 ± 10.6 years; mean height: 1.78 ± 0.12 m; mean weight: 74.5 ± 7.5 kg) participated in the study. The diagnosis of PPV was based on the diagnostic criteria suggested by Brandt (1). Mean duration of the symptoms was 1.3 ± 0.6 years. Patients underwent a standardized diagnostic procedure: Afferent somatosensory deficits were excluded by vibrotactile and proprioceptive assessment. Vestibular testing included a caloric irrigation of the horizontal semicircular canals (30/44 °C) and the head impulse testing of the horizontal vestibulo-ocular reflex (23). These procedures revealed normal somatosensory and vestibular functions in all participants. Anxiety was reported or admitted on direct questioning by eight patients (40%), whereas vegetative symptoms such as palpitations, sweating, dyspnea, or diarrhea were only present in two patients (10%). A history of coexisting panic disorder and/or agoraphobia was obtained in only one patient (5%), whereas four patients (20%) had a confirmed history of depression or anxiety disorder. Nine patients (45%) reported an avoidance behavior of triggering situations, i.e., open spaces, closed places like elevators, crowds, or bridges. None of the patients admitted a regular use of benzodiazepines, antidepressants, or other neuromodulatory drugs. Six patients received physiotherapeutic interventions in history. However, a continued balance training, which could affect the posturography measurements, was not undertaken by any of the participants.

All subjects gave their written informed consent prior to the experiments. The study protocol was approved by the local Ethics Committee. The study was conducted in conformity with the Declaration of Helsinki.

### Measurement of postural sway

**Figure 1:**
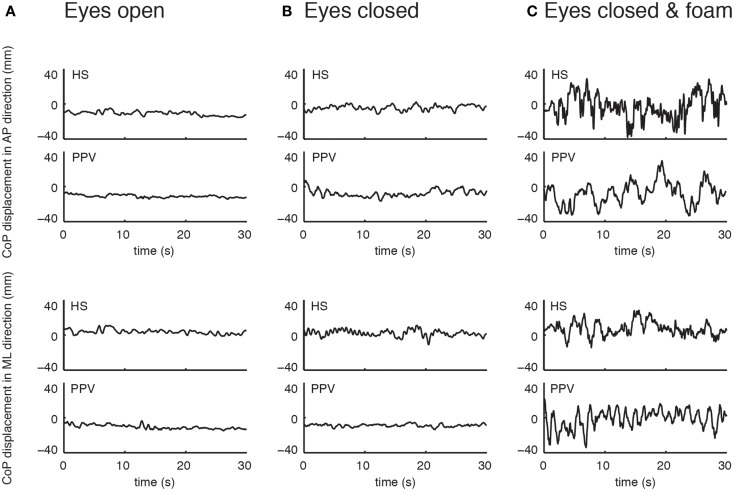
**Example of time series of center of pressure displacement in the anterior-posterior (AP) direction (upper panel) and the medial-lateral (ML) direction (lower panel) for one patient with phobic postural vertigo (PPV) and one healthy subject (HS) with eyes open (A), eyes closed (B), and eyes closed while standing on a foam (C)**.

Static postural control was measured during upright stance on a stabilometer platform (Type 9261 A; Kistler, Winterthur, Switzerland), which transduces force changes exerted on foot support at a sampling frequency of 40 Hz. Subjects were instructed to remain quiet upright stance (feet splayed at an angle of 30 °, arms in hanging position) to omit any voluntary movements during the recordings. The postural control of each subject was recorded for three different stance conditions, standing with eyes open (EO), standing with EC, and standing with eyes closed on a foam rubber (ECF). Each stance condition was recorded for 30 s. The recorded signals were bidirectionally filtered (second-order low-pass Butterworth filter, cut-off frequency of 12.5 Hz) to eliminate low amplitude measurement noise (14). Then the displacement of CoP in the medial-lateral (ML) and the anterior-posterior (AP) directions were calculated (Figure 1). All analysis was done using Matlab (The Mathworks, Version 2011a).

### Data analysis

Nonlinear measures were applied to estimate the structure of postural variability, i.e., the scaling behavior (scaling exponent α) and the signal regularity (SEn) of the CoP time series.

#### Scaling exponent

The scaling exponent α was calculated by using a fractal analysis method for biological time signals called detrended fluctuation analysis (DFA) (24). In a first step, the mean is subtracted from the original time series, which is then integrated:

$$y\left(k\right)=\sum_{i=1}^{k}\left[x\left(i\right)-\bar{x}\right]$$

This integration step transforms the bounded series (i.e., CoP series) into an unbounded series, which has been suggested to avoid shortcomings of earlier methods (25). The integrated series is then divided into windows of equal length *n* ranging from 4 to *N*/4 data points. The local trend of each window *y_n_* is obtained and subtracted from the summed series by using a least-squared fit to obtain the detrended fluctuation *F*(*n*):

$$F\left(n\right)=\sqrt{\frac{1}{N}\sum_{k=1}^{N}{\left[y\left(k\right)-y_{n}\right]}^{2}}$$

**Figure 2:**
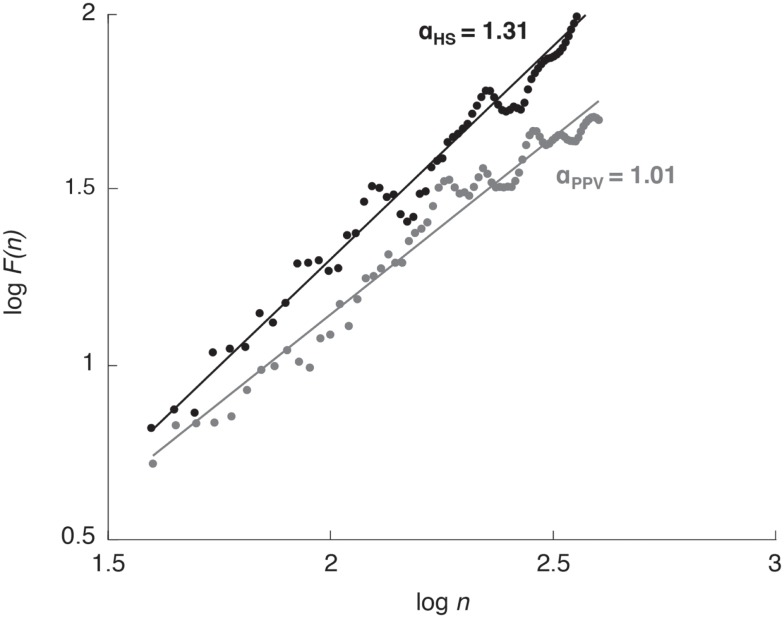
**Detrended fluctuation analysis plot for one healthy subject (HS; black dots and line) and one patient with phobic postural vertigo (PPV; gray dots and line) while standing with eyes open**. The scaling exponent α quantifies the strength of long-range correlations within the CoP time series.

The fluctuation of the detrended series, *F*(*n*), is characterized by the power law *F*(*n*) ∝ *n*^α^, where α is the slope of a double logarithm plot of *F*(*n*) vs. *n* (Figure 2). The scaling exponent α gives a quantitative measure for the strength of long-range correlations within the time series. A scaling exponent α = 0.5 indicates uncorrelated data (i.e., white noise); α values between 0.5 and 1.0 indicate persistent long-range power-law correlations; the closer α is to 1.0 the greater the influence of the distant past when compared with the influence of the recent past. The case of α = 1.0 corresponds to 1/*f* noise, where present events are approximately equally correlated with events from the recent and the very distant past. For α > 1.0 correlations exist but cease to be of a power-law form; α = 1.5 indicates brown noise, i.e., integrated white noise. Brown noise is influenced by the recent past much more strongly than by the distant past and is therefore characterized by only local correlations (24, 26, 27).

#### Sample entropy

The signal regularity was quantified using the SEn analysis (28, 29). The SEn analysis indexes the regularity of a time series by calculating the probability that having repeated itself for a window length *m* within a tolerance of *r*, will also repeat itself for *m* + 1 data points, without allowing self-matches. Thereby, lower values of SEn are associated with higher regularity of the time series, which means a greater likelihood that sets of matching epochs in a time series will be followed by another match within a certain tolerance.

Prior data processing comprised the computation of the increment of the recorded CoP time series according to the suggestions of Govindan et al. (30). The increment data was simply obtained by computing the differences between consecutive CoP values, thereby removing non-stationarity of the original CoP data. After the preceding data processing, SEn values were computed of the increment time series with input parameters *m* and *r* by using the PhysioToolkit-PhysioNetSampEn software (31). Parameters *m* and *r* were chosen according to the procedure described in Ramdani et al. (32), obtaining the value *m* = 3 and *r* = 0.4 for both ML and AP directions.

### Statistical analysis

The effects for each dependent variable were analyzed using a two-way ANOVA with group and condition as factors. *Post hoc* analysis was carried out using a Bonferroni *post hoc* test and results are reported significant if *p* < 0.05. Statistical analysis was performed using SPSS (Version 20).

## Results

The results of the two-way ANOVA are presented in Table 1.

**Table 1:** **Results of the two-way ANOVA on the scaling exponent α and SEn of the CoP displacement in the medial-lateral (ML) and the anterior-posterior (AP) plane**.

	Group (PPV/HS)	Condition (EO/EC/ECF)	Group × condition
**ML DIRECTION**			
Scaling exponent	*F*_1, 40_ = 2.3, *p* = 0.129	***F*_2, 40_ = 3.5**, ***p* = 0.034**	*F*_2, 40_ = 2.0, *p* = 0.135
Sample entropy	***F*_1, 40_ = 33.8**, ***p* < 0.001**	***F*_2, 40_ = 39.2**, ***p* < 0.001**	***F*_2, 40_ = 3.5**, ***p* = 0.033**
**AP DIRECTION**			
Scaling exponent	***F*_1, 40_ = 5.8**, ***p* < 0.001**	***F*_2, 40_ = 1.4**, ***p* = 0.002**	***F*_2, 40_ = 6.4**, ***p* = 0.002**
Sample entropy	***F*_1, 40_ = 30.0**, ***p* < 0.001**	***F*_2, 40_ = 34.1, *p* = 0.001**	*F*_2, 40_ = 3.0, *p* = 0.052

*Significant differences are marked bold*.

*PPV, patients with phobic postural vertigo; HS, healthy subjects; EO, eyes open; EC, eyes closed; ECF, eyes closed and standing on foam*.

### Scaling exponent α

**Figure 3:**
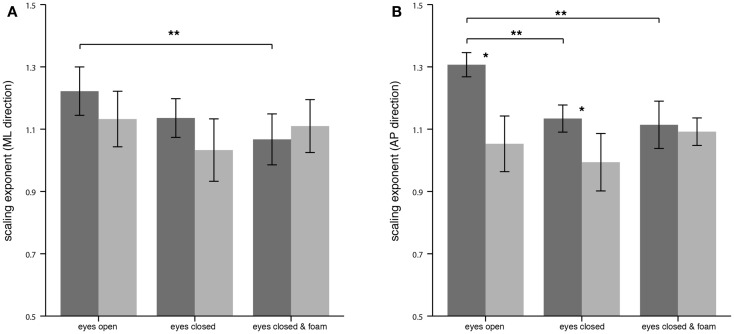
**Scaling exponent α of the center of pressure displacement in the medial-lateral (ML) direction (A) and in the anterior-posterior (AP) direction (B) for healthy controls (*n* = 20; dark gray) and patients (*n* = 20; light gray)**. Asterisks denote significant differences: (*) indicates *p* < 0.05, (**) indicates *p* < 0.01.

For the CoP displacement in the ML direction, no significant differences of α between PPV patients and HS could be detected as reflected in the mean values for EO (PPV vs. HS: 1.13 ± 0.19 vs. 1.22 ± 0.17), for EC (PPV vs. HS: 1.03 ± 0.21 vs. 1.14 ± 0.13) and for ECF (PPV vs. HS: 1.11 ± 0.18 vs. 1.07 ± 0.17). In HS, α was significantly lower for the condition ECF compared to EO (Bonferroni, *p* = 0.009) (Figure 3A).

For the CoP displacement in the AP direction, α of patients compared to HS was significantly lower for the condition EO (PPV vs. HS: 1.05 ± 0.19 vs. 1.31 ± 0.08; *p* < 0.001), and for EC (PPV vs. HS: 0.99 ± 0.20 vs. 1.13 ± 0.09; *p* = 0.007). During standing under ECF no significant differences between the groups were found (PPV vs. HS: 1.09 ± 0.09 vs. 1.11 ± 0.16; *p* > 0.05). In HS, the scaling exponent α was significantly lower for the condition EC as well as for the condition ECF compared to EO (Bonferroni, for both *p* < 0.001). No significant differences of α between the standing conditions were found in PPV patients (Figure 3B).

### Sample entropy

**Figure 4:**
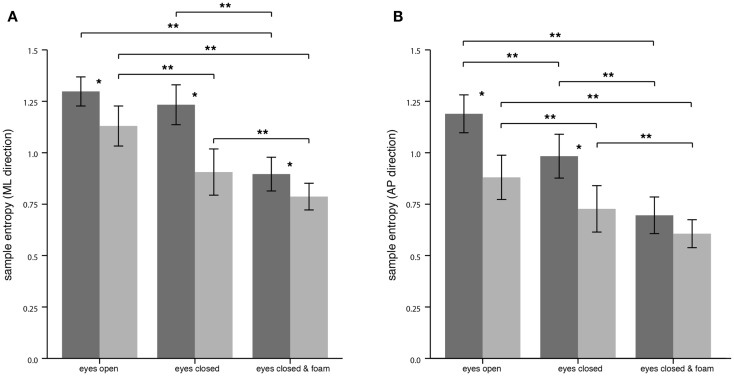
**Sample entropy of the center of pressure displacement in the medial-lateral (ML) direction (A) and in the anterior-posterior (AP) direction (B) for healthy controls (*n* = 20; dark gray) and patients (*n* = 20, light gray)**. Asterisks denote significant differences: (*) indicates *p* < 0.05, (**) indicates *p* < 0.01.

For the CoP displacement in the ML direction, SEn of PPV patients compared to HS was significantly lower for all conditions. The mean values were for EO (PPV vs. HS: 1.13 ± 0.21 vs. 1.30 ± 0.15; *p* = 0.006), for EC (PPV vs. HS: 0.91 ± 0.24 vs. 1.23 ± 0.21; *p* < 0.001) and for ECF (PPV vs. HS: 0.79 ± 0.14 vs. 0.90 ± 0.18; *p* = 0.035). Within the HS group, SEn of the CoP displacement in the ML direction was significantly lower for the condition ECF compared to EO and compared to EC (Bonferroni, both *p* < 0.001). In patients with PPV, SEn in the ML direction was significantly lower for the condition EC as well as for the condition ECF compared to EO [Bonferroni, *p* = 0.011 (EC vs. EO) and *p* < 0.001 (ECF vs. EO)] and significantly lower for the condition ECF compared to EC (Bonferroni, *p* < 0.001) (Figure 4A).

For the CoP displacement in the AP direction, SEn of PPV patients was significantly lower for the conditions EO and EC compared to HS. Mean values were for EO (PPV vs. HS: 0.88 ± 0.23 vs. 1.19 ± 0.20; *p* < 0.001), for EC (PPV vs. HS: 0.73 ± 0.24 vs. 0.98 ± 0.23; *p* = 0.001) and for ECF (PPV vs. HS: 0.61 ± 0.15 vs. 0.70 ± 0.19; *p* > 0.05). Within the HS group, SEn of the CoP displacement pattern was significantly lower for the condition EC as well as for the condition ECF compared to EO [Bonferroni, *p* = 0.007 (EC vs. EO) and *p* < 0.001 (ECF vs. EO)] and for ECF compared to EC (Bonferroni, *p* < 0.001). Within the PPV group, SEn in the AP direction was significantly lower for the condition EC as well as for the condition ECF compared to EO [Bonferroni, *p* = 0.003 (EC vs. EO) and *p* < 0.001 (ECF vs. EO)] and significantly lower for the condition ECF compared to EC (Bonferroni, *p* < 0.001) (Figure 4B).

## Discussion

The results of the study show that during normal balance condition postural control in PPV is characterized by a less complex, i.e., more regular and constrained mode of standing compared to HS. This difference largely disappears for more demanding balance tasks. The results can be interpreted in the way that PPV patients use an inappropriate balance strategy at baseline, which is only used by healthy controls for difficult and demanding balance tasks.

### Complexity in postural control in PPV

Healthy postural control under normal stance conditions exhibits highly irregular, complex dynamics that represent interacting regulatory processes, which operate on different time scales (14). Such processes enable the postural system to prepare for reacting to sudden balancing stresses and thereby enhance the overall stability of a standing subject. When the postural control system is perturbed the complex steady-state dynamics may transiently change to a less complex mode of reactive tuning, which is characterized by closed-loop responses that act on relatively short time periods to restore the balance equilibrium (33). An overall decrease of the inherent complexity in the steady-state dynamics is associated with a functional decline of the postural control system resulting in maladaptive responses to perturbations and thereby destabilizing the balance control (34).

Under unperturbed stance conditions postural control in PPV patients in AP direction was characterized by a decreased scaling exponents α close to 1/*f* noise, indicating an increase in strength of long-range correlations. The relatively stronger dependency between the time scales in the CoP signal indicates that a smaller number of independently controllable system elements contribute to the motor output (22, 35, 36). The increased strength of long-range correlations in the sway pattern of patients with PPV may imply a highly constrained postural behavior and decreased local stability (14) in contrast to the flexible and readily adaptable (with respect to changing balance conditions) healthy mode of standing (33). For HS, increasing the demands of the balance task (i.e., EC, ECF) led to a successive decrease of α toward 1/*f* noise (in ML and AP directions) in accordance to previous studies (14–17, 35). In contrast, the scaling exponent α of patients with PPV did not decrease further and was similar to HS under the same condition. This supports the hypothesis that PPV patients use a strategy at baseline that is used normally only for the most demanding balance tasks. The observation that postural behavior in PPV converges to the healthy postural pattern for more demanding balance tasks has been previously described as a characteristic feature of somatoform dizziness syndromes (9).

Sample entropy, which describes the irregularity of the sway pattern, was found to be reduced in patients with PPV for normal stance condition, indicating that the dynamic structure of the posturogram is characterized by a higher regularity (13, 28). Decreased complexity in terms of a more regular sway pattern indicates that the postural behavior is more rigid within repeating patterns thereby loosing adaptability and local stability (13). Increasing the difficulty of the balance task led to a decrease in SEn in HS in accordance to previous studies (14, 32). This decrease was less pronounced in PPV; thereby SEn was similar between patients and controls for demanding tasks (9). Interestingly, a previous study investigating complexity of postural control in young and older adults found changes of the scaling exponent α and the entropy of sway toward different directions (15). While α was closer to 1/*f* noise in the elderly, entropy was found to be increased compared to young adults. However, the authors concluded that the entropy results are inconsistent with the generally acknowledged hypothesis that complexity in the human physiological system decreases with aging (33, 37).

### Postural control strategies of patients with PPV

Previous studies have proposed a relation between the regularity of CoP displacements and the amount of attention spent in the postural control (13, 14, 35, 38). According to this theory the regularity of body sway is positively correlated to the attentional effort (39). The altered postural control dynamics in PPV correspond to the patient’s feeling of instability even on firm ground (19). In previous studies on PPV, it has been hypothesized that increased co-contraction of anti-gravity muscles may cause the altered sway pattern of the patients (20). HS also use co-contraction instead of reciprocal muscle activation when learning a new motor task or when uncertainty exists about the required motor response (40, 41). Co-contraction stabilizes joints and reduces their mechanical sensitivity to external perturbations and thereby shortens the settling time of the system (40). The changes in variability, which we found in PPV, might be compatible with the co-contraction hypothesis (42), but further investigations including EMG recordings of anti-gravity muscles have to be conducted. Our findings might suggest that inappropriate postural control in PPV is caused by a shift of the postural strategy to a more attentional control. The accompanying decrease of complexity might hinder appropriate reactive tuning thereby causing inadequate responses to perturbation (33). In HS similar postural control modes are observed only under demanding balance conditions, like standing on ice or on uneven surfaces.

Studies on the clinical course of PPV have shown the reversibility of the subjective symptoms (19). For stroke patients it has been shown that the regularity of CoP fluctuations decreases during the rehabilitation whereas in parallel the postural stability increases (35). Analysis of CoP fluctuations might be a promising tool for measuring an objective improvement during recovery from PPV. For the future it will be of interest to investigate the sensitivity and specificity of alterations in the scaling exponent α and SEn as a diagnostic tool in PPV.

In conclusion, the results of the present study indicate an impaired postural behavior of patients with PPV predominantly under normal balance conditions. Their balance control appears to be more regular and constrained compared to HS. These changes in balance control might be linked to an anxious control of posture. Analysis of nonlinearities in body sway carries the potential to achieve objective measurements for the diagnosis of PPV.

## Conflict of Interest Statement

The authors declare that the research was conducted in the absence of any commercial or financial relationships that could be construed as a potential conflict of interest.
